# A case of gastrointestinal tuberculosis with unusual manifestations of carcinomatosis

**DOI:** 10.1002/ccr3.2493

**Published:** 2019-10-24

**Authors:** Parvin Abedi, Pezhman Alavinejad, Seyed Jalal Hashemi, Fatemeh Ahmadi

**Affiliations:** ^1^ Department Midwifery Ahvaz Jundishapur University of Medical Sciences Ahvaz Iran; ^2^ Alimentary Tract Research Center Ahvaz Jundishapur University of Medical Sciences Ahvaz Iran; ^3^ Department Infectious Disease Ahvaz Jundishapur University of Medical Sciences Ahvaz Iran

**Keywords:** carcinomatosis, gastrointestinal, gastrointestinal tuberculosis, tuberculosis

## Abstract

Unusual manifestations of TB may include gastrointestinal symptoms such as abdominal pain, constipation, and IVC thrombosis. These symptoms may be misdiagnosed for carcinomatosis. According to our study even with medical advances, the diagnosis of GI TB is still enormously difficult.

## Graphical abstract

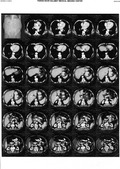

**Abbreviations**
CT: Computerized tomography
GI: Gastrointestinal
IVC: Inferior vena cava
TB: Tuberculosis

## 1 INTRODUCTION

Tuberculosis (TB) is a bacterial disease that is caused by mycobacterium tuberculosis. TB is one of the leading causes of death in the world. According to the World Health Organization, 10 million people were affected by TB in the world in 2017, of whom nearly 1.6 million died from this disease.^1^ According to the WHO country report, the estimated TB incident in Iran has been reported to be 11% in all ages and both sexes in 2017.^2^ TB has two forms of pulmonary and extrapulmonary. While the lungs are the most common site for tuberculosis (85%), the disease may affect any other organs except for nail and hair.^3^ Gastrointestinal tract is one of the organs that could be rarely affected by TB. The symptoms of gastrointestinal TB (GI TB) include nausea, vomiting, change in bowel habit (constipation or diarrhea), and weight loss.^4^ GI TB is caused by the bloodstream from pulmonary tuberculosis, through contaminated food, or by swallowing infected sputum.^5^ The aim of this study was a case presentation of a GI TB with an unusual manifestation of peritoneal carcinomatosis.

## 2 CASE HISTORY

**Figure 1:**
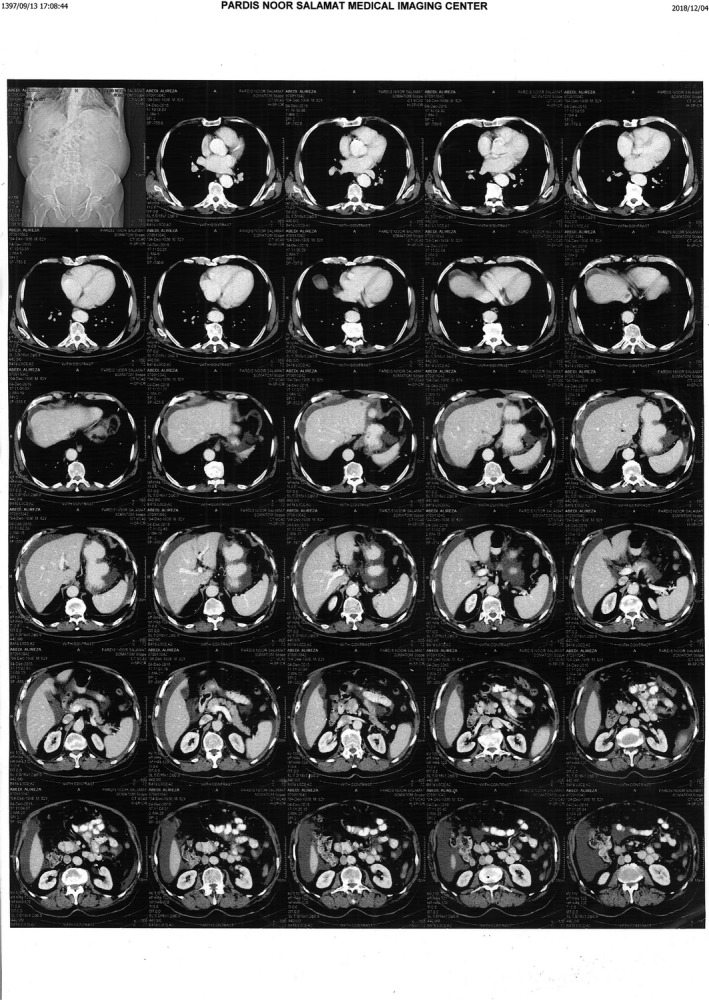
The first CT Scan of the patient on 30/11/2018

The patient is an 82‐year‐old man complaining of abdominal pain and enlargement, constipation, and poor appetite during the last month. He did not have a fever or night sweats. His primary evaluation in an outpatient clinic was done by blood tests, and abdominal CT scan (30/11/2018) and the results revealed some degree of ascites and thrombosis in the inferior vena cava (IVC) (Figure 1). The past medical history of the patient was not significant except for revascularization of coronary heart disease 3 years earlier. He is currently under the following medications for his condition: clopidogrel (75 mg/d), aspirin (80 mg/d), and atorvastatin (20 mg/d). The patient does not have a history of chronic diseases or contact with a person infected with tuberculosis. The patient lost 8 lbs. during the past month.

## 3 DIFFERENTIAL DIAGNOSIS, INVESTIGATIONS, AND TREATMENT

For further evaluation, he referred to a gastroenterologist and on 9/12/2018; he was admitted to the gastroenterology ward. His full laboratory test results including CA125, CA19‐9, CRP, ESR, and ascites fluid tap were determined, and upper endoscopy and colonoscopy were also performed. The endoscopy was normal. While the first colonoscopy performed by the assistant on 10/12/2018 was unsuccessful, the second colonoscopy done by a gastroenterologist on 11/12/2018 was normal. The ascites fluid tap was negative with respect to adenosine deaminase (ADA) that is a TB marker. Because of the thrombosis in the IVC, anticoagulant medication (heparin) was started from 9/12/2018 and continued for a week. The patient's CT scan (14/12/2018) revealed some thickening and nodularity in the perihepatic peritoneum suggesting clinical suspicion of peritoneal carcinomatosis. On 17/12/2018, he underwent a diagnostic laparoscopy and multiple biopsies were taken from the peritoneum. The surgeon's report was metastatic carcinomatosis, which had spread across the peritoneum.

## 4 OUTCOME AND FOLLOW‐UP

The result of ascites fluid analysis was low serum‐ascites albumin gradient (SAAG). The results of blood tests are shown in Table 1. From the clinical view point, the surgeon believed that the patient has carcinoma. The final diagnosis of pathology revealed chronic granulomatous inflammation, and the pathologist recommended PCR for mycobacterium TB (MTB) complex and non‐MTB complex on the specimen. In the next step, PCR was done for the detection of TB and non‐MTB. The results were surprising, and none of the tests was positive for the mycobacterium tuberculosis complex DNA and nontuberculosis mycobacterium DNA except positive serum QuantiFERON test. The Mantoux test was not performed for the patient.

**Table 1:** Results of blood tests

Variables	Results of the blood tests (1/12/2018)	Blood tests after 4 wks treatment
CEA (ECL)	2.87	_
CA 19.9 (ECL) (U/mL)	27.63	_
CA125 (ECL) (U/mL)	446.10	_
QuantiFERON‐TB Gold Plus Results	Positive	_
WBC (10*3/µL)	3.9	4
RBC (10*3/µL)	4.64	4.94
HGB (g/dL)	13.8	13.8
HCT (%)	38.8	43.2
MCV (FL)	83.6	87
MCH (pg)	29.7	27.9
MCHC(g/dL)	35.6	31.9
Platelet 10*3/uL	222	150
RDW (%)	14.1	14.8
ESR	20	_
UREA (mg/dL)	28	
Creatinine (mg/dL)	1	_
SGOT (U/L)	21	27
SGPT (U/L)	10	22
Alkalin Phosphatase (IU/L)	134	128
Total bilirubin (mg/dL)	_	0.30
Direct bilirubin (mg/dL)	_	0.14
Indirect bilirubin (mg/dL)	_	0.2
P.T	_	12.5
TNR	_	1
Partial thromboplastin time (seconds)	_	28
Platelet distribution width (PDW) (%)	_	10
Procalcitonin test PCT (ng/mL)	_	0.102

In compliance with a consultation with an infectious disease specialist, the anti‐TB medications were started. Four weeks after the onset of the medication administration, the clinical symptoms of the patient were generally resolved. The results of the blood tests are presented in Table 1. The patient started to gain weight, and his physician recommended continuation of his anti‐TB medication for up to 6 months. Sputum culture at the beginning of the anti‐TB medications and 4 weeks later was negative.

## 5 DISCUSSION

This is the case of 82‐year‐old man diagnosed with GI TB with the manifestation of peritoneal carcinomatosis. Although GI TB mostly involves peritoneum and intestines, other parts of GI organs such as stomach, rectum, anus, gall bladder, and pancreas could also be involved.^6^ The diagnosis of GI TB is difficult, and delay in diagnosis could potentially cause complications for the patient.^7^ One of the ways of diagnosing GI TB is laparoscopy and biopsy. The granulomatous appearance can be seen in the histopathological examination.^8^ A review by Shi et al^9^ including 85 cases with GI TB in China showed that about half of the patients had coexisting pulmonary TB and only 23.5% of the cases were diagnosed by histopathological examination. Ye et al^10^ showed that granulomatous structure was the most prevalent feature in the intestinal TB. On the other hand, patients with abdominal TB have an increased risk of developing inferior vena cava thrombosis which can cause hepatic and other systemic thromboembolic complications.^11^, ^12^ In the present case study, the primary feature of GI TB was peritoneal carcinomatosis. Laparoscopy was used for diagnosis, and the definitive diagnosis was confirmed by histological examination.

## 6 CONCLUSION

An unusual manifestation of TB includes gastrointestinal symptoms such as abdominal pain, constipation, and IVC thrombosis. These symptoms may be misdiagnosed for carcinomatosis. The results of imaging and blood tests for tumor marker about TB may be misleading. Our study showed that even with medical advances, the diagnosis of GI TB is still a demanding undertaking.

## CONFLICT OF INTEREST

The authors declare that they have no competing interests.

## AUTHOR CONTRIBUTIONS

PA, PA, SJH and FA: were contributed to the conception of the study, data collection and interpretation of data. PA and PA: wrote the first draft of manuscript. All authors checked the manuscript for important intellectual content and approved the final version of manuscript.

## ETHICAL APPROVAL

The informed written consent was obtained from the patient.

## CONSENT FOR PUBLICATION

Written informed consent was obtained from the patient for publication of this case report and any accompanying images. A copy of the written consent is available for review upon the request of Editor‐in‐Chief of this journal.

## Data Availability

None declared.

## References

[1] World Health Organization . Tuberculosis. https://www.who.int/news-room/fact-sheets/detail/tuberculosis. Accessed September 18, 2018.

[2] Data are as reported to WHO . Estimates of TB and MDR‐TB burden are produced by WHO in consultation with countries. 2019.

[3] Haas DW , Des Prez RM . Mycobacterium tuberculosis In: Mandell GL, Bennett JE, Dolin R eds. Mandell, Douglas, and Bennett's Principles and Practice of Infectious Diseases (5th edn). New York, NY: Churchill Livingstone; 1995: 2213‐2243.

[4] Sheer TA , Coyle WJ . Gastrointestinal tuberculosis. Curr Gastroenterol Rep. 2003;5:273‐278. 12864956 10.1007/s11894-003-0063-1

[5] Tripathi PB , Amarapurkar AD . Morphological spectrum of gastrointestinal tuberculosis. Trop Gastroenterol. 2009;30:35‐39. 19624086

[6] Malikowski T , Mahmood M , Smyrk T , Raffals L , Nehra V . Tuberculosis of the gastrointestinal tract and associated viscera. Mycobact Dis. 2018;12:1‐8. 10.1016/j.jctube.2018.04.003 PMC6830173 31720391

[7] Debi U , Ravisankar V , Prasad KK , Sinha SK , Kumar A . Abdominal tuberculosis of the gastrointestinal tract: revisited. World J Gastroenterol. 2014;20:14831‐14840. 25356043 10.3748/wjg.v20.i40.14831 PMC4209546

[8] Sharma MP , Bhatia V . Abdominal tuberculosis. Indian J Med Res. 2004;120:305‐315. 15520484

[9] Shi XC , Zhang LF , Zhang YQ , Liu XQ , Fei GJ . Clinical and laboratory diagnosis of intestinal tuberculosis. Chin Med J. 2016;129:1330‐1333. 27231171 10.4103/0366-6999.182840 PMC4894044

[10] Ye Z , Lin Y , Cao Q , He Y , Xue L. Granulomas as the most useful histopathological feature in distinguishing between crohn's disease and intestinal tuberculosis in endoscopic biopsy specimens. Medicine. 2015;94:e2157. 26656343 10.1097/MD.0000000000002157 PMC5008488

[11] Majid Z , Soomro GB , Bux A , Mandhwani R , Luck NH , Mubarak M . Inferior vena cava thrombosis in a patient with abdominal tuberculosis. Acta Persica Pathophysiol. 2018;10:3.

[12] Jain D , Singh H , Kiran B , Dahiya S , Jain P . Disseminated tuberculosis manifesting as hepatic abscess and IVC thrombosis: a rare presentation. Erciyes Med J. 2018;40(2):103‐107.

